# Supplementary material for: Access to General Practitioners during the COVID-19 pandemic in Portugal—A survey study of patient experiences in an urban setting
Source: PLoS One. 2023 May 23;18(5):e0285899. doi: 10.1371/journal.pone.0285899 (PMC10204959; doi:10.1371/journal.pone.0285899)
Supplement: S1 Appendix — (PDF) [file pone.0285899.s001.pdf]

Questionnaire

Accessibility to the General Practitioner

We seek to know your experiences and your opinion on the accessibility to your General Practitioner, especially during the COVID-19 pandemic.

This questionnaire is voluntary and takes around 15 minutes to fill in. This is a study for a PhD thesis that will also inquire General Practitioners [more information about the study follows on the attached leaflet]. This study has been submitted and approved by the Ethics Review Board of Matosinhos Local Health Unit.

Any queries about the study or its results can be addressed to the research team.

Email: up201707630@med.up.pt

Telephone: 912185922 or 222061820

There are no right nor wrong answers, but it is very important that you answer bearing in mind your most common experiences in the past 6 months.

Whenever you have a health problem, what do you do?

- I always, or almost, see the same doctor
- The doctor I see varies
- Usually, I don't see any doctor
- Usually, I don't have health problems
- Other option, which?

If you always, or almost, see the same doctor, who is he?

- Public sector General practitioner
- Private sector doctor
- A friend or a relative
- Other option, which?

If you see always, or almost, the same doctor, why do you see this doctor and not any other? (you may choose more than one option)

- I get an appointment quickly
- visit hours are convenient
- it is cheap
- it is near me
- he knows me and/or my health conditions
- I trust him
- I have no other choice
- Other option, which?

Why do you see different doctors when you have health problems? (you may choose more than one option)

- I attend the same facility and the doctor that sees me may vary
- I see the doctor that gives me the earliest appointment
- I see the doctor that gives me the most convenient visit hours
- depending on the health problem I see a specialist on the condition
- I like getting different opinions
- depends on the problem being urgent or routine
- depends on my financial situation
- I travel and change address often
- other reason, which?

Why don't you see a doctor when you have a health problem?

- I don't know where to go
- I don't have the time
- I can't afford transports
- I can't afford any appointments, tests or treatments
- I can't find doctors or surgeries where they speak my language
- I call the national phone line
- I do self-care
- other reason, which?

Have you ever used the family practice you are registered in?

- No
- Yes

About your family practice

If you used more than one family practice for the past 6 months, please answer considering the one you have used more often

Usually, how long does it take you to make it to your family practice?

- Up to 10 minutes
- 11 to 20 minutes
- 21 to 30 minutes
- 11 to 60 minutes
- Over 60 minutes

Regarding your family practice facilities, what do you think about…
[very poor/poor/fair/good/very good]

- The front desk
- Waiting rooms
- Consultation rooms
- Entrance and hallways

When you request in family practice an urgent appointment (for yourself or for a relative), usually do you get it…

- On the same day?
- In 1 or 2 working days?
- In 3 to 5 working days?
- After more than 5 working days?
- Usually I don't request urgent appointments

When you request in family practice a routine appointment (for yourself or for a relative), usually do you get it…

- In 1 week?
- In 2 to 3 weeks?
- In 4 to 8 weeks?
- In 9 to 12 weeks?
- After more than 3 months?
- Usually I don't request routine appointments

When you request in family practice a home visit (for yourself or for a relative), usually do you get it…

- In 1 working day?
- In 2 or 3 working days?
- In 4 or 5 working days?
- After more than 5 working days?
- Usually I don't request home visits

Over the past 6 months, how many times did you see your GP (be it for yourself or as a carer of a relative who sees the same GP)?

- not once
- 1 time
- 2 or 3 times
- 4 times or more

When you have an appointment in your family practice, usually how much do you have to wait in the waiting room after the scheduled time?

- I am seen at the scheduled time or even before that
- I wait up to 15 minutes
- I wait between 16 and 30 minutes
- I wait between 31 and 60 minutes
- I wait more than 60 minutes

How do you rate the waiting time for…
[never tried/very poor/poor/fair/good/very good]

- an urgent appointment?
- a routine appointment?
- a routine appointment in the waiting room?
- a home visit?

Are you registered with a GP in your family practice? If so, for how long are you registered with this GP?

- no
- yes, for less than 1 year
- yes, between 1 and 4 years
- yes, between 5 and 10 years
- yes, for more than 10 years

About remote contacts, over the past 6 months
with the front desk, by telephone or e-mail or on the patient portal

Regarding medical prescriptions, how easy or difficult do you find the use of…
[never used/very difficult/difficult/easy/very easy]

- paper scripts?
- scripts sent by text message?
- scripts sent by e-mail?

Regarding the patient portal, how easy or difficult do you find it for…
[never used/very difficult/difficult/easy/very easy]

- booking appointments with your GP?
- request repeat prescriptions?
- insert data on your health summary?

For how long do you usually wait when you request (for yourself or for a relative)…
[never tried/1 working day/2 or 3 working days/4 or 5 working days/more than 5 working days/I have tried but never got it]

- to speak with your GP over the phone?
- a reply to an e-mail?
- a video consultation?
- repeat prescriptions at the front desk?
- repeat prescriptions on the patient portal?
- a remote medical report?
- remote review of test results?

How do you rate the waiting time when you make a request (for yourself or for a relative)…
[never tried/very poor/poor/fair/good/very good]

- to speak with your GP over the phone?
- a reply to an e-mail?
- a video consultation?
- repeat prescriptions at the front desk?
- repeat prescriptions on the patient portal?
- a remote medical report?
- remote review of test results?

What is your opinion about your GP and your family practice for the past 6 months and regarding…
(please choose the option that best describes your opinion. If the question is not applicable in your case, please select 'not applicable/not relevant')
[1 = poor/2/3/4/5 = excellent/not applicable/not relevant]

- easiness of booking a suitable appointment in your family practice?
- easiness of getting through to the practice on the phone?
- easiness of getting through to your GP on the phone?
- time in the waiting room?
- quickness with which urgent problems get sorted?

How do you compare your GP and your family practice before and after the pandemic and regarding…
[no difference/was better before the pandemic/ was worse before the pandemic/Cannot compare]

- easiness of booking a suitable appointment in your family practice?
- easiness of getting through to the practice on the phone?
- easiness of getting through to your GP on the phone?
- time in the waiting room?
- quickness with which urgent problems got sorted?

Over the past 6 months, how often have you attended (for yourself or for a relative) a private doctor?

- not once
- 1 time
- 2 or 3 times
- 4 or 5 times
- 6 times or more

Over the past 6 months, how often have you attended (for yourself or for a relative) the emergency department of a public hospital?

- not once
- 1 time
- 2 or 3 times
- 4 or 5 times
- 6 times or more

Over the past 6 months, have you been admitted to a hospital (staying overnight)?

- no
- yes

About you

What is your gender?

- female
- male

How old are you?

____ years

What is the highest level of education you have completed?

- 3rd grade or less
- 4th grade
- 6th grade
- 9th grade
- 12th grade
- university graduation

Which best describes your employment situation?

- employed
- full time student
- working and studying
- unemployed/job seeker
- disabled for work
- keeping house and/or family caring
- retired
- other situation: ___________

What is your marital status?

- unmarried
- married/living in common law
- divorced/separated
- widowed

Excluding yourself, how many people usually live in your household?

- 0 (I live by myself)
- 1 person
- 2 persons
- 3 persons
- 4 persons
- 5 persons or more
- I live in a nursing home

In your household, are you a parent or legal representative of child under 18?

- no
- yes

In your household, are you the carer of someone with prolonged health issues?

- no
- yes

How often do you use the following devices?
[never/seldom/sometimes/often/daily]

- landline phone
- mobile with no internet connection
- mobile with internet connection/smartphone
- computer or tablet connected to the internet at work/school/university
- computer or tablet connected to the internet at home

How would you rate your general health?

- excellent
- very good
- good
- fair
- poor

Please state if, over the past 6 months, you have suffered from any of the following:
[yes/no]

- asthma (including allergic asthma)
- chronic bronchitis, chronic obstructive pulmonary disease or emphysema
- high blood pressure, that is, hypertension
- osteoarthrosis, or degenerative joint disease
- low back pain or other chronic back problems
- neck pain or other chronic neck problems
- diabetes, excluding during pregnancy
- depression?

Over the past 6 months did you have any of the following severe impairment or disability? [yes/no]

- deafness or severe hearing impairment
- blindness or severe sight impairment
- severe gait impairment
- severe emotional or psychological issue

How many different medicines do you usually take? (consider those you take at least once a month and also those you buy over the counter)

____________ different medicines
