# Supplementary material for: Access to General Practitioners during the COVID-19 pandemic in Portugal—A survey study of patient experiences in an urban setting
Source: PLoS One. 2023 May 23;18(5):e0285899. doi: 10.1371/journal.pone.0285899 (PMC10204959; doi:10.1371/journal.pone.0285899)
Supplement: S1 Table — OR: Odds Ratio; IC: confidence intervals; SD: sociodemographics (sex, age, marital status and education); GP: General Practitioner. *health variables; years registered with the same General Practitioner; self-perceived health status. bold: statistically significant. (PDF) [file pone.0285899.s002.pdf]

S1 Table. Odds Ratio of reporting response over maximum waiting times when requesting a non-urgent consultation.

		**OR** [99%IC]		
		**crude**	**adjusted SD**	**adjusted SD+health***
**sex**	female			
	male	0.63 [0.40-1.00]	0.71 [0.44-1.14]	0.71 [0.43-1.16]
**age**	<40			
	40-54	1.19 [0.65-2.19]	1.27 [0.67-2.41]	1.18 [0.61-2.31]
	55-64	1.32 [0.65-2.69]	1.49 [0.70-3.15]	1.35 [0.61-3.00]
	65-74	0.73 [0.37-1.47]	0.96 [0.43-2.13]	0.90 [0.38-2.11]
	≥ 75	1.07 [0.47-2.46]	1.29 [0.50-3.30]	1.08 [0.40-2.92]
**marital status**	married			
	unmarried	1.53 [0.96-2.45]	1.43 [0.87-2.36]	1.46 [0.88-2.44]
**education**	≤ 4th			
	6th or 9th	0.97 [0.48-1.98]	0.93 [0.43-2.04]	1.02 [0.46-2.30]
	11th or 12th	1.07 [0.56-2.03]	1.01 [0.47-2.17]	1.12 [0.50-2.52]
	university	1.40 [0.74-2.63]	1.27 [0.60-2.69]	1.43 [0.65-3.18]
**years with same GP**	0-<1			
	1-4	0.57 [0.26-1.26]	0.50 [0.22-1.18]	0.49 [0.20-1.16]
	5-10	0.57 [0.26-1.23]	0.45 [0.19-1.02]	0.43 [0.18-1.02]
	>10	0.56 [0.27-1.15]	0.51 [0.23-1.11]	0.48 [0.21-1.07]
**self-perceived health status**	poor			
	fair	1.10 [0.50-2.43]	1.08 [0.46-2.50]	1.01 [0.43-2.38]
	good	1.03 [0.47-2.25]	0.87 [0.37-2.07]	0.87 [0.36-2.10]
	very good	0.86 [0.38-1.92]	0.65 [0.26-1.62]	0.65 [0.26-1.64]
**prescriptions by text message**	difficult			
	easy	0.52 [0.19-1.42]	0.48 [0.16-1.47]	0.50 [0.16-1.55]
**prescriptions by e-mail**	difficult			
	easy	0.36 [0.09-1.36]	0.20 [0.05-1.12]	0.21 [0.04-1.23]
**book appointment on patient portal**	difficult			
	easy	**0.28 [0.13-0.60]**	**0.23 [0.10-0.58]**	**0.24 [0.09-0.61]**
**request prescriptions on patient portal**	difficult			
	easy	**0.23 [0.08-0.70]**	**0.21 [0.05-0.78]**	**0.18 [0.04-0.74]**
**insert data on patient portal**	difficult			
	easy	**0.26 [0.09-0.80]**	**0.20 [0.05-0.90]**	**0.18 [0.04-0.95]**

OR: Odds Ratio; IC: confidence intervals; SD: sociodemographics (sex, age, marital status and education); GP: General Practitioner

*health variables; years registered with the same General Practitioner; self-perceived health status

bold: statistically significant
