# Supplementary material for: Access to General Practitioners during the COVID-19 pandemic in Portugal—A survey study of patient experiences in an urban setting
Source: PLoS One. 2023 May 23;18(5):e0285899. doi: 10.1371/journal.pone.0285899 (PMC10204959; doi:10.1371/journal.pone.0285899)
Supplement: S2 Table — OR: Odds Ratio; IC: confidence intervals; SD: sociodemographics (sex, age, marital status and education); GP: General Practitioner. *health variables; years registered with the same General Practitioner; self-perceived health status. bold: statistically significant. (PDF) [file pone.0285899.s003.pdf]

S2 Table. Odds Ratio of reporting response over maximum waiting times when requesting an urgent consultation.

		OR		
		**crude**	**adjusted SD**	**adjusted SD+health***
**sex**	female			
	male	0.97 [0.59-1.60]	1.25 [0.59-2.67]	1.06 [0.61-1.86]
**age**	<40			
	40-54	1.16 [0.59-2.27]	1.60 [0.66-3.87]	1.27 [0.60-2.69]
	55-64	1.72 [0.79-3.77]	1.47 [0.52-4.15]	2.09 [0.87-5.03]
	65-74	1.07 [0.50-2.31]	1.37 [0.39-4.89]	1.38 [0.50-3.75]
	≥ 75	1.25 [0.51-3.05]	1.28 [0.17-9.89]	1.67 [0.52-5.40]
**marital status**	married			
	unmarried	0.74 [0.44-1.22]	0.78 [0.36-1.68]	0.69 [0.39-1.23]
**education**	≤ 4th			
	6th or 9th	0.86 [0.41-1.80]	0.85 [0.21-3.38]	1.08 [0.44-2.65]
	11th or 12th	1.04 [0.53-2.05]	1.40 [0.38-5.24]	1.68 [0.69-4.11]
	university	1.78 [0.88-3.59]	1.94 [0.50-7.58]	**2.71 [1.06-6.90]**
**years with same GP**	0-<1			
	1-4	1.20 [0.49-2.94]	1.25 [0.48-3.21]	1.23 [0.47-3.25]
	5-10	0.72 [0.31-1.68]	0.85 [0.35-2.09]	0.90 [0.36-2.28]
	>10	0.53 [0.24-1.16]	0.64 [0.28-1.46]	0.61 [0.26-1.43]
**self-perceived health status**	poor			
	fair	0.62 [0.27-1.39]	0.54 [0.23-1.27]	0.54 [0.23-1.30]
	good	0.54 [0.24-1.22]	0.41 [0.16-1.03]	0.41 [0.16-1.03]
	very good	0.82 [0.35-1.97]	0.65 [0.24-1.77]	0.65 [0.23-1.81]
**prescriptions by text message**	difficult			
	easy	1.31 [0.51-3.40]	1.45 [0.51-4.15]	1.57 [0.54-4.61]
**prescriptions by e-mail**	difficult			
	easy	0.42 [0.09-1.91]	0.29 [0.05-1.64]	0.30 [0.05-1.79]
**book appointment on patient portal**	difficult			
	easy	0.81 [0.36-1.84]	0.83 [0.32-2.12]	0.83 [0.31-2.25]
**request prescriptions on patient portal**	difficult			
	easy	0.31 [0.09-1.04]	0.25 [0.05-1.18]	0.23 [0.05-1.17]
**insert data on patient portal**	difficult			
	easy	0.33 [0.09-1.18]	0.29 [0.06-1.36]	0.34 [0.07-1.69]

OR: Odds Ratio; IC: confidence intervals; SD: sociodemographics (sex, age, marital status and education); GP: General Practitioner

*health variables; years registered with the same General Practitioner; self-perceived health status

bold: statistically significant
