# Supplementary material for: Access to General Practitioners during the COVID-19 pandemic in Portugal—A survey study of patient experiences in an urban setting
Source: PLoS One. 2023 May 23;18(5):e0285899. doi: 10.1371/journal.pone.0285899 (PMC10204959; doi:10.1371/journal.pone.0285899)
Supplement: S3 Table — OR: Odds Ratio; IC: confidence intervals; SD: sociodemographics (sex, age, marital status and education); GP: General Practitioner. *health variables; years registered with the same General Practitioner; self-perceived health status. bold: statistically significant. (PDF) [file pone.0285899.s004.pdf]

S3 Table. Odds Ratio of reporting response over maximum waiting times when requesting a repeat prescription at the front desk.

		OR		
		**crude**	**adjusted SD**	**adjusted SD+health***
**sex**	female			
	male	1.07 [0.62-1.85]	1.01 [0.56-1.80]	1.10 [0.60-2.02]
**age**	<40			
	40-54	0.88 [0.35-2.24]	0.77 [0.29-2.05]	0.59 [0.21-1.71]
	55-64	1.51 [0.58-3.96]	1.36 [0.50-3.74]	0.93 [0.31-2.81]
	65-74	1.07 [0.41-2.76]	0.83 [0.29-2.42]	0.54 [0.16-1.75]
	≥ 75	1.45 [0.54-3.90]	1.10 [0.36-3.39]	0.76 [0.22-2.58]
**marital status**	married			
	unmarried	0.80 [0.45-1.42]	0.78 [0.42-1.46]	0.68 [0.35-1.30]
**education**	≤ 4th			
	6th or 9th	0.79 [0.37-1.70]	0.82 [0.36-1.84]	0.93 [0.39-2.22]
	11th or 12th	0.62 [0.30-1.28]	0.66 [0.29-1.52]	0.90 [0.36-2.24]
	university	0.80 [0.39-1.67]	0.89 [0.39-2.04]	1.33 [0.53-3.36]
**years with same GP**	0-<1			
	1-4	0.46 [0.17-1.22]	0.38 [0.14-1.07]	0.37 [0.13-1.07]
	5-10	0.57 [0.24-1.38]	0.57 [0.23-1.44]	0.58 [0.22-1.52]
	>10	0.49 [0.21-1.12]	0.45 [0.19-1.08]	**0.38 [0.15-0.94]**
**self-perceived health status**	poor			
	fair	0.61 [0.28-1.31]	0.62 [0.27-1.39]	0.56 [0.25-1.29]
	good	0.45 [0.20-1.00]	0.43 [0.18-1.05]	**0.39 [0.15-0.97]**
	very good	**0.31 [0.12-0.80]**	**0.26 [0.08-0.80]**	**0.24 [0.08-0.75]**
**prescriptions by text message**	difficult			
	easy	0.53 [0.20-1.39]	0.57 [0.20-1.58]	0.55 [0.19-1.56]
**prescriptions by e-mail**	difficult			
	easy	0.24 [0.05-1.11]	0.26 [0.05-1.25]	0.24 [0.05-1.25]
**book appointment on patient portal**	difficult			
	easy	**0.24 [0.09-0.62]**	**0.14 [0.04-0.46]**	**0.13 [0.03-0.50]**
**request prescriptions on patient portal**	difficult			
	easy	**0.17 [0.05-0.55]**	**0.18 [0.05-0.62]**	**0.11 [0.03-0.47]**
**insert data on patient portal**	difficult			
	easy	0.28 [0.07-1.06]	0.23 [0.04-1.39]	0.11 [0.01-1.24]

OR: Odds Ratio; IC: confidence intervals; SD: sociodemographics (sex, age, marital status and education); GP: General Practitioner

*health variables; years registered with the same General Practitioner; self-perceived health status

bold: statistically significant
