# Supplementary material for: Access to General Practitioners during the COVID-19 pandemic in Portugal—A survey study of patient experiences in an urban setting
Source: PLoS One. 2023 May 23;18(5):e0285899. doi: 10.1371/journal.pone.0285899 (PMC10204959; doi:10.1371/journal.pone.0285899)
Supplement: S4 Table — OR: Odds Ratio; IC: confidence intervals; SD: sociodemographics (sex, age, marital status and education); GP: General Practitioner *health variables; years registered with the same General Practitioner; self-perceived health status. bold: statistically significant. (PDF) [file pone.0285899.s005.pdf]

S4 Table. Odds Ratio of reporting response over three working days when contacting the GP by telephone.

		OR		
		**crude**	**adjusted SD**	**adjusted SD+health***
**sex**	female			
	male	0.68 [0.35-1.35]	0.76 [0.37-1.57]	0.74 [0.34-1.60]
**age**	<40			
	40-54	0.89 [0.36-2.22]	0.94 [0.36-2.46]	0.81 [0.29-2.29]
	55-64	0.91 [0.32-2.62]	0.91 [0.31-2.71]	0.68 [0.20-2.29]
	65-74	0.84 [0.30-2.34]	0.85 [0.26-2.84]	0.53 [0.13-2.07]
	≥ 75	1.28 [0.45-3.64]	1.03 [0.29-3.62]	0.88 [0.22-3.47]
**marital status**	married			
	unmarried	1.50 [0.77-2.93]	1.31 [0.62-2.76]	1.19 [0.53-2.68]
**education**	≤ 4th			
	6th or 9th	0.48 [0.18-1.28]	0.47 [0.16-1.36]	0.61 [0.20-1.89]
	11th or 12th	0.54 [0.22-1.33]	0.54 [0.19-1.55]	0.74 [0.23-2.34]
	university	0.82 [0.33-2.08]	0.78 [0.26-2.33]	1.28 [0.39-4.26]
**years with same GP**	0-<1			
	1-4	0.32 [0.10-1.01]	**0.22 [0.07-0.77]**	**0.21 [0.06-0.75]**
	5-10	0.36 [0.11-1.12]	**0.29 [0.08-0.97]**	0.29 [0.08-1.04]
	>10	**0.33 [0.12-0.94]**	**0.30 [0.10-0.90]**	**0.25 [0.08-0.79]**
**self-perceived health status**	poor			
	fair	0.69 [0.26-1.82]	0.75 [0.27-2.14]	0.63 [0.22-1.87]
	good	0.53 [0.19-1.47]	0.52 [0.16-1.66]	0.45 [0.13-1.50]
	very good	0.36 [0.12-1.06]	**0.27 [0.08-0.97]**	**0.24 [0.06-0.89]**
**prescriptions by text message**	difficult			
	easy	0.45 [0.15-1.38]	0.70 [0.21-2.39]	0.74 [0.21-2.64]
**prescriptions by e-mail**	difficult			
	easy	**0.01 [0.01-0.69]**	**0.11 [0.01-0.89]**	**0.07 [0.01-0.71]**
**book appointment on patient portal**	difficult			
	easy	**0.32 [0.11-0.93]**	0.31 [0.09-1.12]	0.26 [0.06-1.06]
**request prescriptions on patient portal**	difficult			
	easy	**0.11 [0.02-0.53]**	**0.05 [0.01-0.50]**	**0.02 [0.00-0.27]**
**insert data on patient portal**	difficult			
	easy	0.24 [0.05-1.15]	0.17 [0.02-2.02]	0.03 [0.00-2.64]

OR: Odds Ratio; IC: confidence intervals; SD: sociodemographics (sex, age, marital status and education); GP: General Practitioner

*health variables; years registered with the same General Practitioner; self-perceived health status

bold: statistically significant
