# Supplementary material for: Access to General Practitioners during the COVID-19 pandemic in Portugal—A survey study of patient experiences in an urban setting
Source: PLoS One. 2023 May 23;18(5):e0285899. doi: 10.1371/journal.pone.0285899 (PMC10204959; doi:10.1371/journal.pone.0285899)
Supplement: S5 Table — OR: Odds Ratio; IC: confidence intervals; SD: sociodemographics (sex, age, marital status and education); GP: General Practitioner *health variables; years registered with the same General Practitioner; self-perceived health status. bold: statistically significant. (PDF) [file pone.0285899.s006.pdf]

S5 Table. Odds Ratio of reporting response over three working days when contacting the GP by e-mail.

		OR		
		**crude**	**adjusted SD**	**adjusted SD+health***
**sex**	female			
	male	1.23 [0.70-2.15]	1.46 [0.80-2.65]	1.30 [0.70-2.42]
**age**	<40			
	40-54	0.92 [0.46-1.83]	0.95 [0.46-1.93]	0.93 [0.44-2.00]
	55-64	0.89 [0.39-2.04]	0.88 [0.37-2.05]	0.81 [0.32-2.07]
	65-74	1.00 [0.42-2.38]	0.86 [0.33-2.26]	0.88 [0.31-2.48]
	≥ 75	0.86 [0.30-2.46]	0.56 [0.16-1.93]	0.50 [0.13-1.89]
**marital status**	married			
	unmarried	1.31 [0.76-2.26]	1.43 [0.80-2.57]	1.32 [0.71-2.43]
**education**	≤ 4th			
	6th or 9th	0.57 [0.22-1.51]	0.46 [0.16-1.33]	0.55 [0.18-1.67]
	11th or 12th	0.57 [0.24-1.36]	0.44 [0.16-1.22]	0.66 [0.22-2.00]
	university	0.85 [0.38-1.90]	0.68 [0.26-1.78]	0.99 [0.34-2.84]
**years with same GP**	0-<1			
	1-4	0.61 [0.25-1.52]	**0.22 [0.07-0.77]**	0.63 [0.25-1.60]
	5-10	**0.27 [0.11-0.69]**	**0.29 [0.08-0.97]**	**0.29 [0.11-0.73]**
	>10	**0.31 [0.13-0.73]**	**0.30 [0.10-0.90]**	**0.30 [0.13-0.72]**
**self-perceived health status**	poor			
	fair	0.55 [0.22-1.39]	0.60 [0.22-1.64]	0.63 [0.23-1.76]
	good	0.40 [0.16-1.00]	0.41 [0.15-1.14]	0.44 [0.16-1.26]
	very good	0.43 [0.17-1.08]	0.38 [0.13-1.13]	0.38 [0.13-1.16]
**prescriptions by text message**	difficult			
	easy	0.55 [0.20-1.54]	0.56 [0.19-1.67]	0.55 [0.18-1.71]
**prescriptions by e-mail**	difficult			
	easy	**0.15 [0.04-0.57]**	**0.10 [0.02-0.45]**	**0.11 [0.02-0.52]**
**book appointment on patient portal**	difficult			
	easy	0.46 [0.19-1.08]	0.43 [0.17-1.12]	0.47 [0.17-1.29]
**request prescriptions on patient portal**	difficult			
	easy	0.35 [0.12-1.03]	0.40 [0.12-1.38]	0.41 [0.11-1.52]
**insert data on patient portal**	difficult			
	easy	0.34 [0.11-1.11]	0.33 [0.09-1.21]	0.31 [0.06-1.28]

OR: Odds Ratio; IC: confidence intervals; SD: sociodemographics (sex, age, marital status and education); GP: General Practitioner

*health variables; years registered with the same General Practitioner; self-perceived health status

bold: statistically significant
