# Supplementary material for: Access to General Practitioners during the COVID-19 pandemic in Portugal—A survey study of patient experiences in an urban setting
Source: PLoS One. 2023 May 23;18(5):e0285899. doi: 10.1371/journal.pone.0285899 (PMC10204959; doi:10.1371/journal.pone.0285899)
Supplement: S6 Table — GP: General Practitioner. (PDF) [file pone.0285899.s007.pdf]

S6 Table. Participant's comparison of EUROPEP accessibility dimensions between pandemic and pre-pandemic times.

	got worse during pandemic	sees no difference
	%	
getting suitable appointment n=438	50.5	43.2
getting through to practice on phone n=499	50.3	44.7
being able to speak to GP on phone n=345	38.6	55.9
time waiting room n=464	24.4	59.1
quick services urgent health problems n=445	37.8	55.1

GP: General Practitioner
